# A Pilot Study on the Role of Computed Tomography in the Management of Patients with Coronary Artery Anomalies in Romania

**DOI:** 10.3390/jcdd10040170

**Published:** 2023-04-15

**Authors:** Adriana Sorina Capisizu, Dragos Cuzino, Silviu Marcel Stanciu

**Affiliations:** 1 Faculty of General Medicine, Carol Davila University of Medicine and Pharmacy, 8 Eroii Sanitari Bvd, 050474 Bucharest, Romania; 2 Clinical Radiology-Medical Imaging Center, Dr. Carol Davila Central Military Emergency University Hospital, 134 Calea Plevnei Str., 010825 Bucharest, Romania; 3 Center for Cardiovascular Diseases, Laboratory of Noninvasive Cardiovascular Functional Explorations, Dr. Carol Davila Central Military Emergency University Hospital, 134 Calea Plevnei Str., 010825 Bucharest, Romania

**Keywords:** coronary artery anomalies, computed tomography angiography

## Abstract

Coronary artery anomalies may occur during embryogenesis and can lead to changes in the vascularization of the heart, possible ischemia, and an increased risk of sudden death. A retrospective study was conducted with the aim of assessing the prevalence of coronary anomalies in a Romanian sample of patients, investigated with computed tomography angiography for coronary artery disease. The objectives of the study were to identify the anomalies of the coronary arteries and to conduct an anatomical classification according to Angelini. The study also consisted of evaluations regarding coronary artery calcification in the sample of patients by the Agatston calcium score and assessments regarding the presence of cardiac symptoms and their association with coronary abnormalities. The results showed a prevalence of coronary anomalies of 8.7%, of which 3.8% were origin and course anomalies and 4.9% were coronary anomalies with intramuscular bridging of the left anterior descending artery. Recommendations for practice include the widespread use of coronary computed tomography angiography for the diagnosis of coronary artery anomalies and coronary artery disease in larger patient groups and encouraging this investigation across the country.

## 1. Introduction

The coronary arteries (CA) are blood vessels that originate in the aorta and provide vascularization to the heart muscle. Abnormalities may occur during embryogenesis and can lead to changes in the anatomy and function of the coronary arteries [1]. The prevalence of coronary artery anomalies (CAA) varies in the scientific literature between 1% and 5.6% [2]. This is due to the use of heterogeneous nomenclature and variable diagnostic methods, namely, coronary angiography and autopsies. The widespread use of coronary computed tomography angiography (CCTA) has led to a higher prevalence of coronary artery anomalies being reported (i.e., 7.9%) [2,3,4]. CAAs are often underdiagnosed because they are not routinely tested for, so early diagnosis is important as it could prevent cases of myocardial ischemia in the area corresponding to the anomaly and, even, sudden death. As such, nowadays, CCTA is considered the gold standard for the diagnosis and characterization of CAA [2,5,6,7].

The variable incidence of CAA in the scientific literature is also due to inconsistencies in the definition of anomalous variants. Angelini proposed several criteria for establishing what is considered normal anatomy; if this is excluded, the rest of the variants can be considered abnormalities [8]. There is also a numerical expression for some features, such as variations encountered in more than 1% of the general unselected population, but there are other cases where a qualitative description is required [5,8]. Variations refer to simple variants in coronary anatomy, such as the type of coronary dominance, early branching, or the presence of the intermediate branch [9]. CAAs can also be classified functionally as ischemia obligatory or non-ischemic [8]. There are some considerations; for example, intramuscular bridges are included in several CAA classifications, although from a strictly epidemiological point of view they are found in 0.5% to 12% of the population [2]. Other abnormalities, such as separate origins of the LAD and LCX or an acute take-off angle, vary in incidence but generally have no hemodynamic consequences and are considered benign in most cases [2,5,8,9].

According to 2020 ESC guidelines and 2018 AHA/ACC guidelines for adults with congenital anomalies, CCTA is used for the diagnosis and anatomical evaluation of CAA [10,11]. For patient management, each coronary anomaly specificity must be considered, especially in high-risk anatomy, including ostial abnormalities with acute take-off angles, abnormal courses between large vessels, and intramural trajectories. Additionally, the presence of cardiac symptoms and functional investigation evidence of stress-induced ischemia contribute to recommendations for limiting sports activities and surgical correction [8,9].

To establish the possible causes of ischemia, the association of coronary atheromatous infiltration was evaluated by calculating the calcium score. Correlations between the CCTA result and the cardiological examination were sought, as was the association between the presence of CAA and the presence of cardiac symptoms.

In Europe, a first national multicenter study on CAA, MuSCAT in the Netherlands, started in 2020 and has been ongoing for the last three years, involving six centers and an established working protocol that included CCTA [12]. Other studies reported a prevalence of CAA of 7.9% in a single center in Zurich that investigated 1759 patients by CCTA over a four-year period [3]. Angelini et al. found a prevalence of 5.64% in a continuous series of 1950 coronary angiographies [7].

Regarding CAA in Romania, the current level of knowledge is limited, and the national prevalence has not been established. This study is important because, to our knowledge, it is the first to study the prevalence of CAA by CCTA in a southeastern region. However, a study conducted in the northwestern region of the country reported a prevalence of CAA of 10.3% in patients investigated by the CCTA over a period of 3.3 years [13]. Other CAA prevalence data were obtained by coronary angiography, which found a CAA prevalence of 0.5% [14] and 0.53% [15].

Thus, the aim of the retrospective pilot study was to investigate the prevalence of CAA in patients investigated with CCTA for coronary artery disease (CAD).

The specific objectives of the pilot study were:

1. identifying the characteristics of the Romanian patients with CAA;
2. identifying the particularities of coronary anomalies from an anatomical perspective;
3. establishing the association between CAA and calcium score;
4. determining the association between cardiac symptoms and the presence of coronary anomalies in the sample of patients.

## 2. Materials and Methods

### 2.1. Study Design and Participants

A retrospective study was conducted to establish the prevalence of coronary artery anomalies in a group of patients investigated with CCTA for CAD at Carol Davila Central Military Emergency University Hospital, Bucharest, Romania, between February 2021 and December 2022.

The inclusion criteria for the patients were: adults over 18 years of age with suspected CAD according to clinical assessment by the attending cardiologist, and patients with known CAD to assess clinical disease. The exclusion criteria were serum creatinine >1.2 mg/dl, pregnant patients, patients with a history of iodine allergy, patients with unstable angina pectoris, and patients with acute coronary syndrome (ECG changes, elevated cardiac biomarkers).

For categorizing CAA, an anatomical classification from Angelini was used, which includes four main categories of anomalies: anomalies of origin and course, anomalies of intrinsic coronary arterial anatomy and route, anomalies of coronary termination, and anomalous anastomotic vessels [1,8].

Out of 203 CCTA examinations, 184 were included; the rest were excluded due to poor image quality caused by patient movement, motion artifacts due to incorrect breath holding, or spontaneous heart rate irregularities during the CT examination.

The study was conducted in accordance with the Declaration of Helsinki and approved by the Ethics Committee of Dr. Carol Davila’s Central Military Emergency University Hospital, Bucharest, Romania, No. 433/12.01.2021.

### 2.2. Methodology

All patients underwent a clinical examination and resting electrocardiogram (ECG) prior to CCTA. Coronary anatomy was analyzed by CCTA, and examinations were evaluated for coronary artery origin location, acute angle, height of detachment, course, and intramuscular coronary track length. Coronary artery anatomical variants were established, and coronary anomalies were detected and classified according to Angelini’s CAA classification. A semi-automated Agatston score was used to calculate the degree of coronary artery calcification in all patients. Data was retrieved from the patient’s medical records and digital records on patient registration in the CT console. The presence of typical and atypical cardiac symptoms suggestive of coronary artery disease was established (angina pectoris, chest tightness, palpitations, dyspnea, or decreased effort tolerance).

### 2.3. Medical Investigations

#### 2.3.1. Coronary Computed Tomography Angiography (CCTA)

The technique used for the diagnosis of CAA was coronary CT angiography, which consisted of a 128-detector CT system (Revolution EVO 128-slice, GE Healthcare, Milwaukee, WI, USA) and a dedicated angiographic protocol with retrospective cardiac gating. The protocol includes lateral and anteroposterior scouts. The calcium score was initially assessed, a Smart Prep slice 2 cm below the carina was obtained, and then the contrast acquisition was performed. The scan range was 2 cm below the carina, covering the whole heart to the base. The contrast medium was heated to 37 degrees, and 60 mL of iodinated contrast medium were administered at a rate of 5.5 mL/s, followed by 30 mL of contrast medium mixed with serum and then 30 mL of saline solution. A heart rate below 65 beats per minute was obtained by oral administration of beta-blockers approximately one hour before the examination or intravenously at the beginning and during the examination. For a better visualization of the coronary lumen, two puffs of sublingual nitroglycerin were administered at the beginning of the examination, which helped to relax the smooth muscles in the vascular walls.

For the evaluation of the coronary anatomy, image post-processing on a GE Medical System AW VolumeShare 7 station with Volume Viewer Applications CardIQ Function included automatic first coronal trajectory detection and automatic branch annotation. A 3D volumetric reconstruction was performed. The coronary pathway was validated by a physician who also verified the origin of the trunks and the path of the branches and made corrections and additions to the reconstructed pathway. Common images in axial, sagittal, coronal, and various oblique planes were used for the analysis of coronary origins and routes. Maximum intensity projection (MIP) images were used to identify the vascular anatomy but were limited in the analysis of anatomical details and stenoses. Multiplanar reconstructions (MPR) were analyzed with the possibility of making precise measurements. Multiplanar curve reconstructions (CPR) were also used for vascular lumen analysis with the possibility of continuous visualization of the vessel, even the ones with curbed routes. Lumen measurements and stenosis analysis were also performed. Volume rendering (VR) 3D images were used to analyze the entire vascular tree anatomy.

#### 2.3.2. Resting Electrocardiogram (ECG)

A resting ECG was used to measure the electrical activity of the heart. The rhythmic contractions of the heart were recorded, reflecting the electrical signal generated by the sinoatrial (SA) node, with the ECG signal’s continuous P, Q, R, S, and T peaks at the corresponding moments of the cardiac cycle. The standard electrocardiogram consisted of 12 leads, with correct electrode placement being critical to interpreting the 12-lead resting electrocardiogram. The ECG calibration parameters were set up to record at the standard 25 mm/s and 10 mm/mV. Various cardiac conditions were detected, and ECG changes suggestive of cardiac ischemia were recorded [16,17].

#### 2.3.3. Agatston Calcium Score

The Agatston score is a semi-automated tool used to measure the calcification of coronary arteries. It is routinely performed at the beginning of cardiac CT examinations using a low dose of radiation and an unenhanced CT scan. A density factor is calculated according to the density attenuation in Hounsfield units and the area of the calcification speck. Based on the total value of the calcium score, coronary artery disease (CAD) was graded into five categories: 0 calcium score (no CAD), 1–10 calcium score (minimal CAD), 11–100 (mild), 101–400 (moderate), and more than 400 (severe CAD) [18].

#### 2.3.4. Anatomical Classification of the CAA

CAA were classified using an anatomical classification from Angelini (2007), and the main categories of anomalies included were: anomalies of origin and course, anomalies of intrinsic coronary arterial anatomy and route, anomalies of coronary termination, and anomalous anastomotic vessels [1,8].

### 2.4. Statistical Analysis

Statistical data analysis was performed using IBM SPSS Statistics 20. For the qualitative data, frequencies and percentages were used, whereas for the continuous data, it was the mean and the standard deviation. For the association between the sample of patients with CAA and other qualitative variables, the Chi-square (*x^2^*) test was used. The statistically significant threshold was a *p*-value < 0.05.

## 3. Results

### 3.1. Romanian Patient Characteristics with CAA

The study included 184 individuals who underwent CCTA (87 males and 97 females, mean age 57.05 years). Further, 29 were young patients under 45 years old. The CAAs were present in 16 (8.7%). According to the *x^2^* test, there was no statistically significant association between the age and gender of patients and the presence of CAA.

In the whole sample, there were 88 (47.8%) males and 96 (52.2%) females, while in the patients with CAA, there were 9 (56.2%) females and 7 (43.8%) males. In what concerns the age, there was a threshold division, namely, under 45 years and above 45 years. Thus, in the whole sample, there were 29 (15.8%) persons with an age under 45 years, while in the sample with CAA, the number of patients with an age under 45 years was 2 (12.5%). The vast majority of patients have ages above 45 years, and in the whole sample there were 155 (84.2%) patients within this range, whereas in the sample with CAA there were 14 (87.5%) patients.

### 3.2. The Particularities of Coronary Artery Anatomy, Coronary Artery Anomalies from an Anatomical Perspective

The CAA detected among the study patients were seven (3.8%) anomalies of origin and course and nine (4.9%) anomalies of coronary arterial anatomy with intramuscular bridging. Regarding the normal coronary artery anatomical variants among all patients, 147 (79.9%) presented right coronary artery dominance, 13 (7.1%) presented left coronary artery dominance, and codominance was found in 24 (13.0%) of the patients. The prevalence of the intermediate branch in all patients was 76 (41.3%), while in patients with CAA, the prevalence was 5 (31.3%).

**Figure 1:**
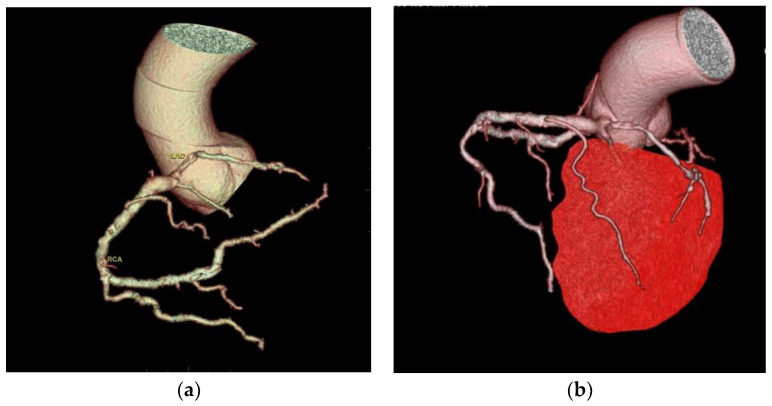
Anomaly of the origin of the left main coronary artery arising from the right anterior sinus. (**a**) CCTA volume rendered 3D images show the vascular tree anatomy, in which an origin anomaly is present. The left main coronary artery arises from the right sinus immediately above the right coronary artery with an anomalous course anteriorly to the left pulmonary artery towards the anterior interventricular sulcus, where it forms the anterior interventricular and circumflex arteries. The right coronary artery is well developed, with increased dimensions and the presence of a well-developed marginal branch and collateral circulation. Diffuse atheromatous infiltration of the left coronary tree is also present; (**b**) CTAA volume rendered 3D images show the vascular tree anatomy in relation to the left ventricle.

The age ranges were between 21 and 80 years for the whole sample, and for the patients with CAA, the age ranges were between 21 and 73 years.The CAAs detected were classified according to Angelini [8] and will be described in more detail as follows:

Two cases of anomalous origin of the left main coronary artery arising from the right sinus were found, as depicted in Figure 1.

**Figure 2:**
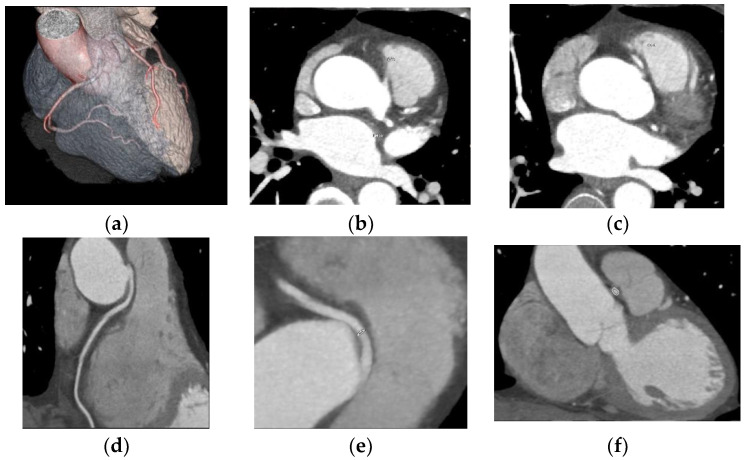
Anomaly of the origin of the right coronary from the left anterior coronary sinus, with a course between the pulmonary artery and the aorta. (**a**) CCTA volume rendered cardiac transparency images showing anomaly of origin and course; the right coronary anomaly originates from the left coronary sinus with an anomalous course between the left pulmonary artery and the aorta. Normal origin of the left main coronary artery from the posterior portion of the left coronary sinus with a normal trajectory. Intermediate filiform branch is present; (**b**) CCTA axial contrast-enhanced image at the aortic root showing anomaly of the origin and course; the right coronary abnormally originates from the left coronary sinus; (**c**) CCTA axial contrast-enhanced image at the aortic root showing RCA with an anomalous course between the pulmonary artery and the aorta; (**d**) CCTA curved planar reformatted image of the RCA; (**e**) CCTA curved planar reformatted image of the RCA origin; (**f**) CCTA vessel cross-section image of the RCA with an anomalous course between the pulmonary artery and the aorta.

One case of right coronary anomaly originating from the left coronary sinus was found, with an anomalous course between the left pulmonary artery and the aorta, according to Figure 2.

**Figure 3:**
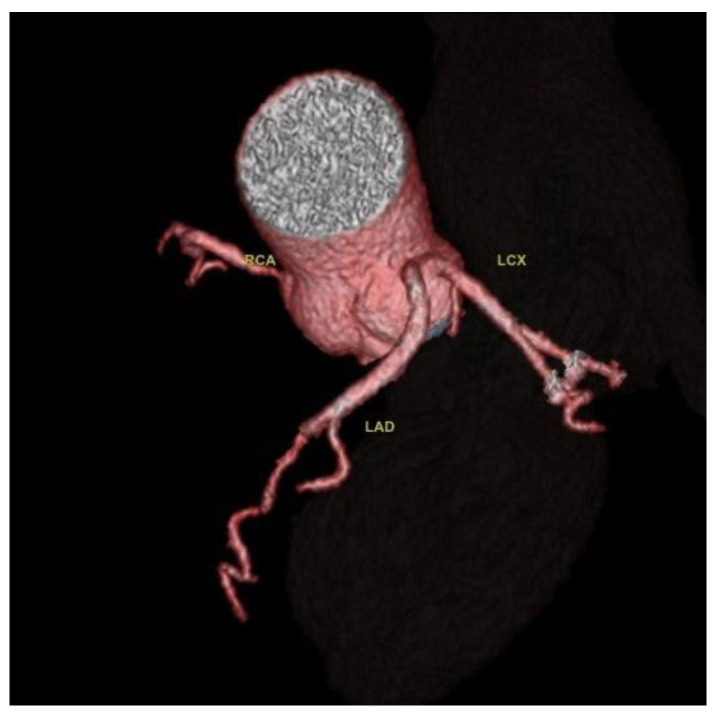
Separate origins of the left anterior descending artery and circumflex artery from the left anterior coronary sinus. CCTA Volume rendered 3D images of the vascular tree anatomy, showing the LAD and LCX arise directly from the left coronary sinus. RCA with normal origin from the right coronary sinus and normal course.

Two cases of separate origins of LAD and LCX arising directly from the left coronary sinus were found, as illustrated in Figure 3.

**Figure 4:**
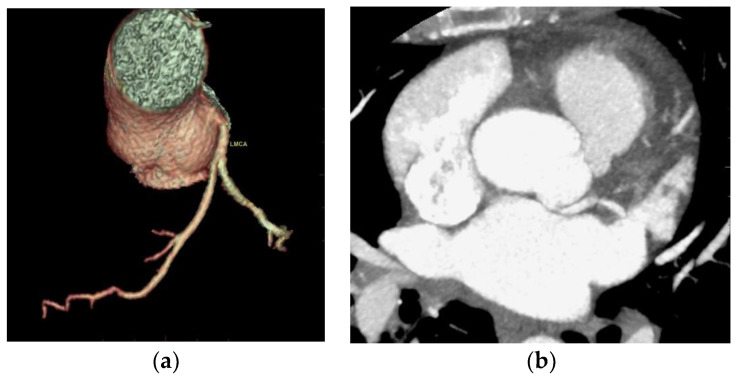
Acute take-off angle of the left main coronary artery. (**a**) CCTA volume rendered 3D images of the vascular tree anatomy show detachment of the left main coronary artery from the upper and posterior portion of the left coronary sinus at a sharp angle; (**b**) CCTA MIP axial contrast-enhanced image at the aortic root shows detachment of the left main coronary artery from the left coronary sinus at a sharp angle with the presence of posterior contact with the left atrium.

One case of detachment of the left main coronary artery from the left coronary sinus at an acute angle was found (Figure 4).

**Figure 5:**
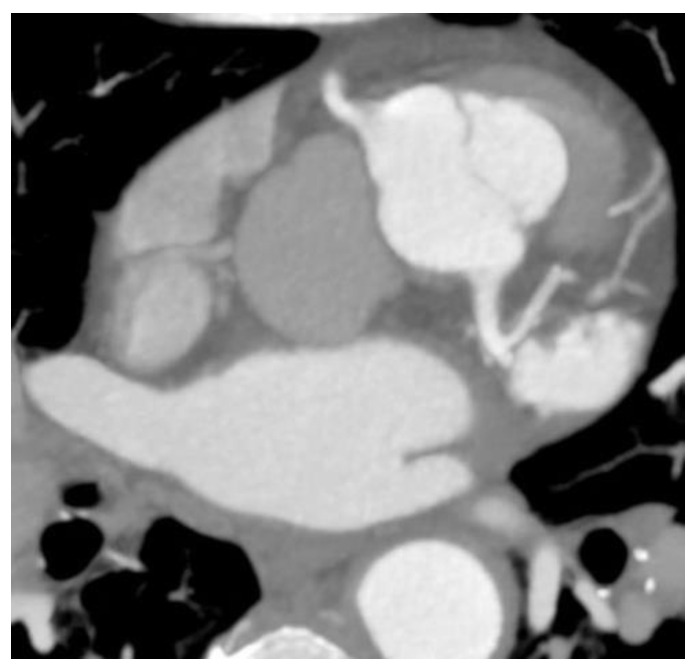
Congenital cardiac malformation with corrected transposition of the great arteries and abnormally rotated aortic cusps and origin of the coronary arteries. CCTA MIP oblique axial contrast-enhanced image at the aortic root shows congenital cardiac malformation with corrected transposition of the great arteries. The aorta is to the left of the pulmonary artery trunk; the aorta is in continuity with the left systemic ventricle, and the pulmonary artery is in continuity with the pulmonary ventricle. The malformation also involves aortic cusps that are abnormally rotated in the axial plane: two anterior sinuses, one on the right and one on the left, related to the patient, and a posterior one. The left main coronary artery arises from the posterior sinus, shows a posterior trajectory of 10 mm, and divides into the LAD and LCX. The LAD arises at a sharp angle and descends on the anterior face of the systemic ventricle. The RCA arises from the anterior right sinus and descends towards the anterior interventricular sulcus.

One case of congenital cardiac malformation with transposition of the great arteries and associated variation of origin and course of the coronary arteries was found (Figure 5).

**Figure 6:**
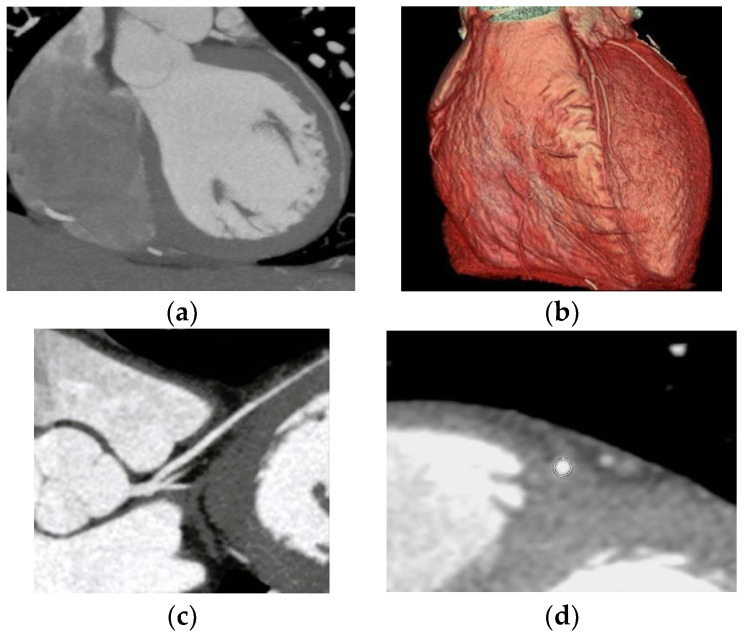
Intramuscular bridging of the left anterior descending artery. (**a**) CCTA oblique sagittal maximum intensity projection image of the LAD route shows intramuscular bridging of the left anterior descending artery in the second segment for a 30 mm distance and 3 mm depth; (**b**) CTAA volume rendered 3D images show the LAD in the atrioventricular groove entering an intramural route; (**c**) CCTA curved planar reformatted image of the LCA entering an intramural route; (**d**) CCTA vessel cross-section image of the LCA with intramural course.

Nine cases of coronary anomalies with intramuscular bridging of the left anterior descending artery were found (Figure 6).

### 3.3. Identifying the Association between CAA and Coronary Calcium Score

Among all patients, 104 (56.5%) presented no calcium load, 6 (3.3%) presented a minimal calcium score (1–10 UA), 26 (14.1%) presented a mild calcium impairment (11–100 UA), 33 (17.9%) presented a moderate calcium score (101–400 UA), and 15 (8.2%) presented a calcium score of more than 400 UA. Among patients with CAA, 13 (81.3%) presented a calcium score of zero, and 3 (18.8%) had moderate calcium impairment. No statistically significant association was detected between the presence of CAA and the levels of calcium, as shown in Table 1.

### 3.4. Determining the Presence of Cardiac Symptoms and the Association with CAA

Among all patients, 94 (51.1%) presented cardiac symptoms, and among patients with CAA, 12 presented cardiac symptoms, and there was a statistically significant association between the presence of cardiac symptoms and CAA (*p* = 0.045).

Regarding the management of patients, the functional aspects of coronary anomalies were analyzed with the help of cardiac tests, mainly resting ECG. Among the study sample of patients, 23 (12.5%) presented changes suggestive of cardiac ischemia in the resting ECG, and among patients with CAA, 3 (18.8%) presented changes suggestive of cardiac ischemia in the resting ECG. In addition, no statistically significant association was detected between the presence of CAA and the presence of changes in the resting ECG (*p* = 0.42).

Among the 16 patients with CAA, 2 underwent revascularization treatment, and the remaining patients were discharged with close follow-up.

## 4. Discussion

The aim of this cross-sectional pilot study was to assess the prevalence of CAA in a Romanian sample of patients who were investigated with CCTA for CAD. The sample of patients with CAA consisted of 16 patients, mainly women with an age higher than 45.

In what concerns the prevalence of coronary anomalies in the scientific literature, it varies between 1% and 5.6%, but the use of CCTA has led to a higher prevalence (7.9%) of CAA being reported [2,3,4]. In this study, the prevalence of coronary artery anomalies obtained was 8.7%, similar to the one found in scientific literature. Among all patients, 7 (3.8%) had anomalies of origin and course, and 9 (4.9%) had anomalies of coronary arterial anatomy with intramuscular bridging.

Two cases of a left coronary artery originating from the right coronary sinus were found; this is a rare anomaly; the prevalence of coronary artery anomalies arising from the opposite site in the scientific literature is between 0.1% and 0.2%, with the left coronary arising from the right sinus being six times rarer than the right coronary artery originating from the left sinus [19]. This is considered an anomaly associated with a risk of sudden death estimated between 0.17% and 0.35% because of the larger myocardial territory associated with ischemic risk [19,20].

One case of CAA with a right coronary anomaly originating from the left coronary sinus and an anomalous course between the pulmonary artery and the aorta was detected; a dynamic compressive effect could not be excluded at this level. According to the anatomical classification of the CAA of Angelini, there are several possible routes for the anomalous location of the coronary ostium at improper aortic sinus: in the posterior atrioventricular groove, retroaortic, between the aorta and the pulmonary artery, intraseptal, anterior to the pulmonary flow, in the posteroanterior interventricular groove [8]. The CAA with a route between the aorta and the pulmonary artery is considered the highest-risk route and is associated with an increased risk of sudden death. Young people are the most vulnerable to sudden death; the risk decreases for middle-aged and elderly people [5,21,22]. One hypothesis for why this effect occurs is that, during exercise, the aortic root and the pulmonary trunk expand and compress the coronary that passes between them. Another hypothesis is that the course of the coronary artery is partially embedded in the aortic wall and is laterally compressed during exertion [5,22,23].

Two cases of separate origins of the LAD and LCX directly from the left coronary sinus were detected. Separate origins of LAD and LCX from the left coronary sinus is considered a benign variant, but some CAA classifications include it. It is considered a common CAA with a frequency between 0.41% and 0.67% [5,24].

One anomaly of detachment of the left main coronary artery from the left coronary sinus at an acute angle was encountered. In terms of route, the normal proximal orientation of the LM and RCA is 45° to 90° to the aortic wall, with a subepicardial extramural route to destination [5]. In scientific literature, the prevalence of acute angles of detachment of the coronary artery from the coronary sinus is 2% [2].

Coronary anomalies may coexist with other cardiac anomalies [1]. For instance, in 26% of cases, coronary anomalies are associated with aortic root anomalies [5]. In this study, one congenital cardiac malformation with corrected transposition of the great arteries was encountered. The CCTA has an increased capacity to diagnose cardiac malformations, coronary anatomy, and their relationship. In patients with transposition of the great vessels, the identification of coronary anatomy is fundamental for patient management and, eventually, for surgical intervention [25,26]. The cardiac malformation in this study is on the benign spectrum of the condition due to the presence of three separate sinuses and no malignant coronary course running between the aortic pathway and the right ventricular outflow tract.

In coronary intramyocardial bridging, a portion of the epicardial coronary pathway is covered by heart muscle; the artery most affected by this condition is the LAD artery [1]. Nine cases of coronary artery intramuscular bridging were detected, and the findings are consistent with the scientific literature; all cases included the left anterior descending artery. Intramuscular bridges are frequently an incidental encounter in CCTA examinations, and only some of them associate systolic compression with a possible reduction in myocardial perfusion. Intramuscular bridges are sometimes associated with atypical angina pectoris. The impact of myocardial bridges on the risk of myocardial infarction is debatable. They are generally considered benign conditions, but the length and depth of the intramyocardial bridge may influence the degree of coronary dysfunction, with some studies showing an association of myocardial bridges with increased risk of myocardial infarction [27,28,29].

Identifying the association between CAA and coronary calcium score involves vessels with abnormalities of origin and route, which have a longer, more tortuous path and flow turbulence that cause damage to the vascular endothelium and tend to trigger the development of atherosclerosis. Thus, CAA of origin and trajectory contribute to myocardial ischemia [5,8,22,24]. In this study, 56.5% of the patients presented no calcium load, 3.3% presented a minimal calcium score, 14.1% presented a mild calcium impairment, 17.9% presented a moderate calcium score, and 8.2% presented a calcium score greater than 400 UA. The calcium score in patients with CAA was zero in 81.3%, and 18.8% had moderate calcium score values; no statistically significant association was detected between the presence of CAA and the presence of calcium levels.

Concerning the presence of cardiac symptoms and the management of patients with CAA, numerous studies have shown that CCTA is the gold standard method for investigating CAA [2,6,7].

There are many advantages to using CCTA to investigate CAA. Compared to coronary angiography, CCTA can provide further information regarding the relationship with other cardiac structures [2,30,31], is non-invasive [3,32], and requires less irradiation [33,34,35]. Furthermore, compared to coronary angiography and MR angiography (MRA), CCTA has better accessibility, time, and cost efficiency [30,31]. In this study, some of the patients were referred because of previous equivocal tests like coronary angiography and echocardiography. The results of several studies have shown that CCTA is a viable non-invasive method for detecting coronary anomalies, including in the case of equivocal coronary angiography [36].

For the management of CAA, international guidelines (2020 ESC guidelines and 2018 AHA/ACC guidelines for adults with congenital anomalies) recommend CCTA for the diagnosis and anatomical evaluation of CAA [10,11]. Under certain conditions, if there are symptoms and evidence of a stress-induced abnormality in association with the abnormality, surgical intervention is recommended, but for asymptomatic patients, surgical intervention is only considered. Special attention should be given to high-risk anatomy, including ostial abnormalities such as an acute take-off angle, a slit-like orifice, an ostium more than 1 cm above the sinotubular junction, and an abnormal course between large vessels that contribute to recommendations for limiting sports activities and surgical correction [10,11].

Thus, the presence of cardiac symptoms plays an important role in deciding the patient’s management. In this study, among all patients, 94 (51.1%) presented cardiac symptoms, and among patients with CAA, 12 presented cardiac symptoms, and there was a statistically significant association between the presence of cardiac symptoms and CAA.

Functional aspects were analyzed with resting ECGs; in the study, 23 (12.5%) of all patients showed changes suggestive of cardiac ischemia, and among patients with CAA 3, 18.8% showed changes.

Among the 16 patients with CAA in the study, 2 underwent revascularization treatment. The remaining individuals were discharged with close follow-up.

There are limitations to the study from an imaging perspective, namely thatthe coronary CT angiography image quality is impaired by the impossibility of performing respiratory commands and by cardiac arrhythmias. There are contraindications and limitations to coronary CT angiography, such as pregnancy, chronic renal failure, or iodine allergy.

Other limitations relate to the pilot study, namely the number of patients. The outcomes of the study cannot be generalized to the entire population of Romania; future directions involve continuing the study on a larger patient group with diverse socio-demographic characteristics.

The increase in the number of CCTA investigations, compared to coronary angiography, leads to an increase in the prevalence of detected coronary anomalies. Currently, there are no randomized multicenter prospective studies in the scientific literature, and the management of patients with CAA is performed by analyzing each individual case, considering symptoms, age, and the presence of risk abnormalities. More evidence based on multicenter studies and follow-up studies is needed to make the current recommendations more effective [37].

## 5. Conclusions

CCTA is a useful anatomical method to identify and characterize coronary anomalies. The prevalence of coronary artery anomalies obtained was 8.7%. According to Angelini’s classification, various coronary anomalies have been detected; among all patients, 3.8% had anomalies of origin and course, and 4.9% had anomalies of coronary arterial anatomy with intramuscular bridging. CCTA also provides information about the presence of atherosclerotic disease; by calculating the calcium score, the atheromatous burden was determined.

## Figures and Tables

**Table 1:** The association between CAA and coronary calcium score.

Variable	Whole Sample (n = 184)	Patients with CAA (n = 16)	
Coronary calcium levels	Frequency (%)	Frequency (%)	*p* value
No calcium	104 (56.5%)	13 (81.3%)	0.17 (NS)
Minimal	6 (3.3%)	0 (0%)	
Mild	26 (14.1%)	0 (0%)	
Moderate	33 (17.9%)	3 (18.8%)	
High	15 (8.2%)	0 (0%)	

Note: NS = not statistically significant.

## Data Availability

The data presented in this study are available on reasonable request from the corresponding author (A.S.C.). The data are not publicly available due to ethical restrictions.

## References

[1] Pérez-Pomares J.M., de la Pompa J.L., Franco D., Henderson D., Ho S.Y., Houyel L., Kelly R.G., Sedmera D., Sheppard M., Sperling S. (2016). Congenital coronary artery anomalies: A bridge from embryology to anatomy and pathophysiology-a position statement of the development, anatomy, and pathology, ESC Working Group. Cardiovasc. Res..

[2] Gentile F., Castiglione V., De Caterina R. (2021). Coronary Artery Anomalies. Circulation.

[3] Ghadri J.R., Kazakauskaite E., Braunschweig S., Burger I.A., Frank M., Fiechter M., Gebhard C., Fuchs T.A., Templin C., Gaemperli O. (2014). Congenital coronary anomalies detected by coronary computed tomography compared to invasive coronary angiography. BMC Cardiovasc. Disord..

[4] Namgung J., Kim J.A. (2014). The prevalence of coronary anomalies in a single centre of Korea: Origination, course, and termination anomalies of aberrant coronary arteries detected by ECG-gated cardiac MDCT. BMC Cardiovasc. Disord..

[5] Villa A.D., Sammut E., Nair A., Rajani R., Bonamini R., Chiribiri A. (2016). Coronary artery anomalies overview: The normal and the abnormal. World J. Radiol..

[6] Manghat N.E., Morgan-Hughes G.J., Marshall A.J., Roobottom C.A. (2005). Multidetector row computed tomography: Imaging congenital coronary artery anomalies in adults. Heart.

[7] Shi H., Aschoff A.J., Brambs H.J., Hoffmann M.H. (2004). Multislice CT imaging of anomalous coronary arteries. Eur. Radiol..

[8] Angelini P. (2007). Coronary artery anomalies: An entity in search of an identity. Circulation.

[9] Rahalkar A.M., Rahalkar M.D. (2009). Pictorial essay: Coronary artery variants and anomalies. Indian J. Radiol. Imaging.

[10] Ruperti-Repilado F.J., Thomet C., Schwerzmann M. (2020). 2020 ESC guidelines on treatment of adult congenital heart disease (ACHD). Heart.

[11] Stout K.K., Daniels C.J., Aboulhosn J.A., Bozkurt B., Broberg C.S., Colman J.M., Crumb S.R., Dearani J.A., Fuller S., Gurvitz M. (2019). 2018 AHA/ACC Guideline for the Management of Adults with Congenital Heart Disease: A Report of the American College of Cardiology/American Heart Association Task Force on Clinical Practice Guidelines. Circulation.

[12] Koppel C.J., Driesen B.W., de Winter R.J. (2021). The first multicentre study on coronary anomalies in the Netherlands: MuSCAT. Neth. Heart J..

[13] Popa L.E., Petresc B., Catana C., Moldovanu C.G., Szep M.B., Rancea R., Molnar A., Buruian M.M. (2019). Prevalence and characteristics of coronary artery anomalies detected by CT angiography in Romanian population. Hum. Vet. Med. Int. J. Bioflux Soc..

[14] Railean E.C., Nedelciu I., Macovei L., Omete G., Arsenescu Georgescu C. (2011). Anomalii de emergenta coronariana: Prevalenta populationala, corelatii clinice si paraclinice. Rev. Soc. Med. Interna.

[15] Predescu L., Bucsa A., Croitoru M., Mereuta A., Platon P., Postu M., Zarma L., Licheardopol L., Deleanu D. (2022). Clinical and Paraclinical Characteristis of Patients with Coronary Artery Anomalies. Rom. J. Cardiol..

[16] Klabunde R.E. (2017). Cardiac electrophysiology: Normal and ischemic ionic currents and the ECG. Adv. Physiol. Educ..

[17] Rahman S., Karmakar C., Natgunanathan I., Yearwood J., Palaniswami M. (2022). Robustness of electrocardiogram signal quality indices. J. R. Soc. Interface.

[18] Goel A., Weerakkody Y., Knipe H., Hacking C., Condon J., Jha P. Agatston Score. Reference Article. https://radiopaedia.org.

[19] Peñalver J.M., Mosca R.S., Weitz D., Phoon C.K. (2012). Anomalous aortic origin of coronary arteries from the opposite sinus: A critical appraisal of risk. BMC Cardiovasc. Disord..

[20] Eckart R., Scoville S., Campbell C., Shry E.A., Stajduhar K.C., Potter R.N., Pearse L.A., Virmani R. (2004). Sudden death in young adults: A 25-year review of autopsies in military recruits. Ann. Intern Med..

[21] Basso C., Maron B.J., Corrado D., Thiene G. (2000). Clinical profile of congenital coronary artery anomalies with origin from the wrong aortic sinus leading to sudden death in young competitive athletes. J. Am. Coll. Cardiol..

[22] Sloan K., Majdalany D., Connolly H., Schaff H. (2008). Inferior wall myocardial infarction caused by anomalous right coronary artery. Can. J. Cardiol..

[23] Fischer H., Hedman A., Öhman T. (2015). Coronary artery anomaly common cause of sudden cardiac death in young people. Unusual diagnosis that requires a targeted investigation in order not to be missed. Lakartidningen.

[24] Young P.M., Gerber T.C., Williamson E.E., Julsrud P.R., Herfkens R.J. (2011). Cardiac imaging: Part 2, normal, variant, and anomalous configurations of the coronary vasculature. AJR Am. J. Roentgenol..

[25] Sasi S.S., Padley P.G., Rubens M.B., Gatzoulis M.A., Siew Y.H., Edward D.N. (2013). Great Vessel and Coronary Artery Anatomy in Transposition and Other Coronary Anomalies: A Universal Descriptive and Alphanumerical Sequential Classification. JACC Cardiovasc. Imaging.

[26] Kumar P., Bhatia M. (2021). Role of Computed Tomography in Postoperative Follow-up of Arterial Switch Operation. J. Cardiovasc. Imaging.

[27] Sternheim D., Power D.A., Samtani R., Kini A., Fuster V., Sharma S. (2021). Myocardial Bridging: Diagnosis, Functional Assessment, and Management. JACC J. Am. Coll. Cardiol..

[28] D’Amario D., Ciliberti G., Restivo A., Laborante R., Migliaro S., Canonico F., Sangiorgi G.M., Tebaldi M., Porto I., Andreini D. (2022). RIALTO Registry Investigators. Myocardial bridge evaluation towards personalized medicine: Study design and preliminary results of the RIALTO registry. Eur. Heart J. Suppl..

[29] Tarantini G., Migliore F., Cademartiri F., Fraccaro C., Iliceto S. (2016). Left Anterior Descending Artery Myocardial Bridging: A Clinical Approach. J. Am. Coll. Cardiol..

[30] Zeina A.R., Blinder J., Sharif D., Rosenschein U., Barmeir E. (2009). Congenital coronary artery anomalies in adults: Non-invasive assessment with multidetector CT. Br. J. Radiol..

[31] Cheezum M.K., Ghoshhajra B., Bittencourt M.S., Hulten E.A., Bhatt A., Mousavi N., Shah N.R., Valente A.M., Rybicki F.J., Steigner M. (2017). Anomalous origin of the coronary artery arising from the opposite sinus: Prevalence and outcomes in patients undergoing coronary CTA. Eur. Heart J. Cardiovasc. Imaging.

[32] Bluemke D.A., Achenbach S., Budoff M., Gerber T.C., Gersh B., Hillis L.D., Hundley W.G., Manning W.J., Printz B.F., Stuber M. (2008). Noninvasive coronary artery imaging: Magnetic resonance angiography and multidetector computed tomography angiography: A scientific statement from the American Heart Association Committee on Cardiovascular Imaging and Intervention of the Council on Cardiovascular Radiology and Intervention, and the Councils on Clinical Cardiology and Cardiovascular Disease in the Young. Circulation.

[33] Husmann L., Valenta I., Gaemperli O., Adda O., Treyer V., Wyss C.A., Veit-Haibach P., Tatsugami F., von Schulthess G.K., Kaufmann P.A. (2008). Feasibility of low-dose coronary CT angiography: First experience with prospective ECG-gating. Eur. Heart J..

[34] Herzog B.A., Wyss C.A., Husmann L., Gaemperli O., Valenta I., Treyer V., Landmesser U., Kaufmann P.A. (2009). First head-to-head comparison of effective radiation dose from low-dose 64-slice CT with prospective ECG-triggering versus invasive coronary angiography. Heart.

[35] Morin R.L., Gerber T.C., McCollough C.H. (2003). Radiation dose in computed tomography of the heart. Circulation.

[36] Datta J., White C.S., Gilkeson R.C., Meyer C.A., Kansal S., Jani M.L., Arildsen R.C., Read K. (2005). Anomalous coronary arteries in adults: Depiction at multi-detector row CT angiography. Radiology.

[37] Gräni C. (2018). Anomalous coronary arteries and the risk of adverse cardiac events. Cardiovasc. Med..

